# Induced ankylosis of a primary molar for skeletal anchorage in the mandible as alternative to mini-implants

**DOI:** 10.1186/s40510-015-0090-0

**Published:** 2015-06-18

**Authors:** Matina V Angelopoulou, Despina Koletsi, George Vadiakas, Demetrios J Halazonetis

**Affiliations:** Division of Pediatric Dentistry, School of Dentistry, Marquette University, 1801 W Wisconsin Ave, 53233 Milwaukee, WI USA; Department of Orthodontics, School of Dentistry, National and Kapodistrian University of Athens, 2 Thivon Str., 11527 Athens, Greece; Department of Paediatric Dentistry, School of Dentistry, National and Kapodistrian University of Athens, 2 Thivon Str., 11527 Athens, Greece

**Keywords:** Induced ankylosis, Molar protraction, Anchorage, Congenitally missing premolars

## Abstract

**Background:**

Mesial protraction of mandibular posterior teeth requires increased anchorage to avoid undesired tooth movements. Orthodontic mini-implants have become a popular and successful way to increase skeletal anchorage in such cases. However, mini-implants may cause injury to adjacent teeth or anatomical structures and may lead to tissue inflammation. Induced ankylosed primary teeth have been used in the past as abutments for the protraction of the maxilla in cases of maxillary retrognathism. However, this technique has not been described in the literature for the protraction of mandibular molars. The aim of this paper is to present, through a case report, an alternative to mini-implant devices to maximize anchorage in the mandible by inducing ankylosis on a primary molar.

**Findings:**

A 13-year-old female with class II right malocclusion, deep bite, and congenitally missing right second premolars was referred for orthodontic treatment. Treatment plan involved removal of the primary teeth and mesial protraction of the posterior. In the mandible, ankylosis was induced on the retained primary second molar by extraction, bisection, replantation of the mesial part after endodontic treatment, and bonding of a rigid splint. Ankylosis was diagnosed after 10 weeks and a closing T-loop sectional wire was inserted to move the permanent first molar mesially. At 6 months, the remaining space was closed using elastic chain on a rectangular stainless steel wire with tip-back bends, supported by class II elastics.

**Conclusions:**

Induced ankylosis of primary teeth can be an alternative to orthodontic mini-implants in selected cases, with minimal risks and maximum biocompatibility.

## Findings

### Clinical examination

**Fig. 1:**
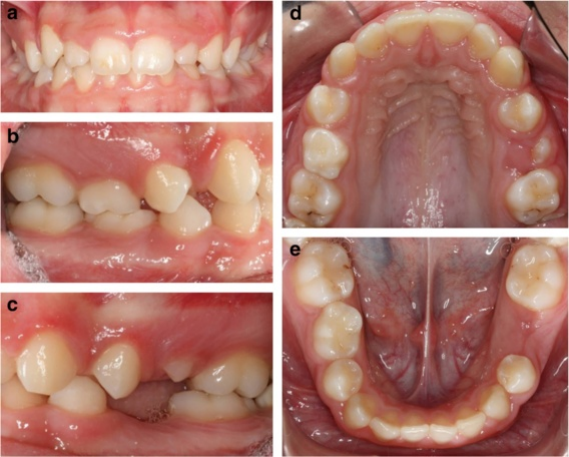
Initial clinical examination of **a** frontal view, **b** right lateral view, **c** left lateral view, **d** maxillary occlusal view, and **e** mandibular occlusal view

A 13-year-old Caucasian female was referred for orthodontic treatment. The patient’s medical history was noncontributory. Extraoral clinical examination revealed a symmetric face, normal proportions of the upper and lower facial height, a slightly convex profile, and an acute nasolabial angle. Intraoral clinical examination showed mild generalized gingivitis with fair oral hygiene and no caries. Evaluation of the occlusion revealed a dental class II molar and canine relationship on the right side and class I molar and canine relationship on the left side, deep bite, slight malalignment of the anterior teeth, and deviation of the mandibular midline to the right by 2 mm. Both maxillary and mandibular right second primary molars were present while all other primary teeth had exfoliated (Fig. 1).

**Fig. 2:**
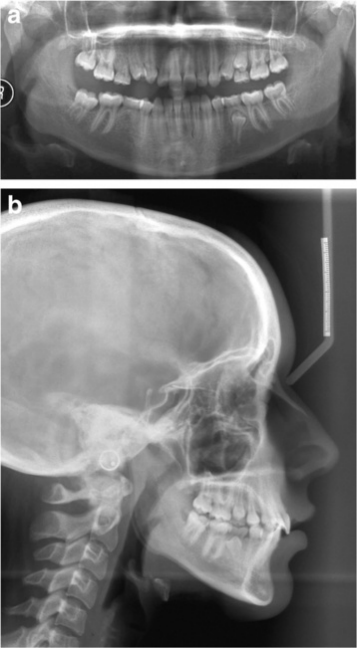
**a** Panoramic radiograph showing congenitally missing right 2nd premolars. **b** Cephalometric radiograph at the start of treatment

Radiographic examination revealed congenitally missing right second premolars and all third molars (Fig. 2a). Cephalometric analysis showed a skeletal class I relationship and hyperdivergent skeletal pattern (Fig. 2b, Table 1).

**Table 1:** Initial and final cephalometric measurements

	Initial	Final
SNA (°)	76.4	76.8
SNB (°)	73.3	72.7
ANB (°)	3.1	4.1
Wits (mm)	0.2	−0.4
SN to GoGn (°)	42.8	42.9
Maxillary incisor to NA (°)	19.8	13.4
Maxillary incisor to NA (mm)	4.1	−0.1
Mandibular incisor to NB (°)	25.5	27.6
Mandibular incisor to NB (mm)	4.4	3.9
Interincisal angle (°)	131.6	134.9
Mandibular incisor to GoGn (°)	89.5	92.0

### Treatment alternatives

Correction of the canine relationship, overbite, and midline discrepancy would be accomplished using fixed appliances. Regarding the agenesis of the second premolars, the patient was offered two choices: maintain the primary molars and prosthetically replace them when they would eventually exfoliate, or extract them and close the space by mesial movement of the first and second permanent molars [1, 2]. The patient decided to follow the second option in order to avoid the need for implants or other prostheses in the future.

Mesial movement of molars is taxing on anchorage, especially in the mandibular arch [3–5]. Considering that the mandibular midline was already shifted to the agenesis side, skeletal anchorage was deemed an efficient option. However, instead of placing a mini-implant [3, 5, 6], we decided to use the primary molar as a biological alternative. We induced ankylosis and used the mesial root as skeletal anchorage. Induced ankylosed primary teeth have been used in the past as abutments for the protraction of the maxilla in cases of maxillary retrognathism [7–9]. However, this technique has not been described in the literature for the protraction of mandibular molars.

### Treatment steps

**Fig. 3:**
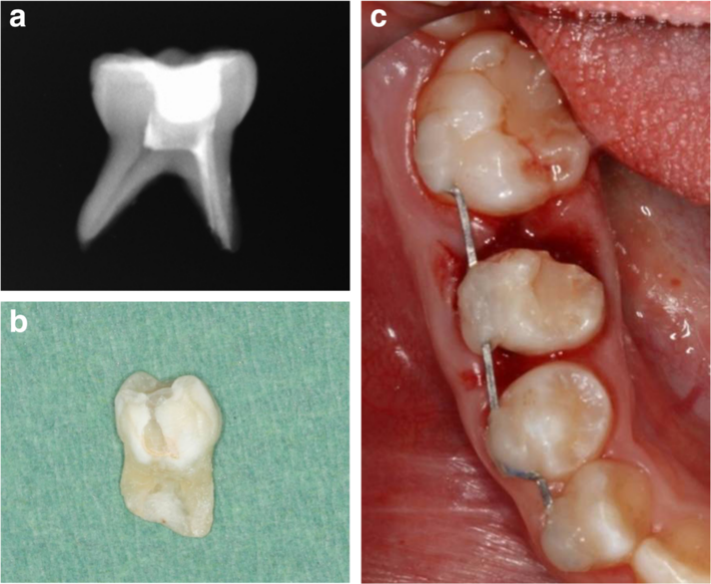
Induced ankylosis technique **a** extraoral root canal treatment of the mesial root of the primary tooth, **b** hemisectomy of the primary tooth, and **c** replantation of the mesial part and splinting

The ankylosis technique included extraction of the primary molar under local anesthesia followed by root canal treatment of the mesial root, performed ex vivo using a zinc oxide and eugenol sealer (Fig. 3a). The crown was then restored with composite resin and a 2-mm apicoectomy and hemisectomy of the tooth was performed (Fig. 3b). To induce ankylosis, we followed the management protocol for avulsed teeth with a closed apex and extraoral dry time exceeding 60 min [10]. Periodontal ligament cells were removed from the root surface with a scaler. Sixty minutes after the extraction of the tooth, the socket was irrigated with saline solution and the mesial root was replanted with slight pressure. To stabilize the tooth and assure ankylosis, a rigid splint was applied, extending from the right canine to the right first molar, using a 0.016″ × 0.022″ stainless steel (SS) rectangular wire (Fig. 3c).

**Fig. 4:**
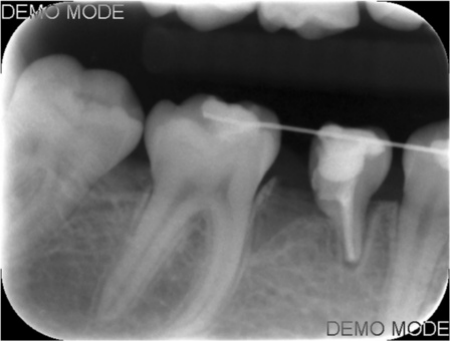
Radiograph 10 weeks post replantation showing the absence of periodontal ligament and root ankylosis

**Fig. 5:**
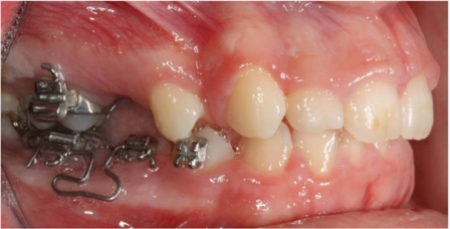
Initial orthodontic space closure using a T-loop to close the space between the primary molar segment and the permanent first molar

**Fig. 6:**
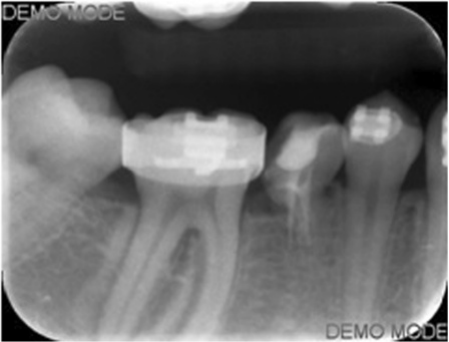
Radiograph 6 months post replantation showing almost total root resorption and complete space closure between the 1st permanent molar and 2nd primary molar

**Fig. 7:**
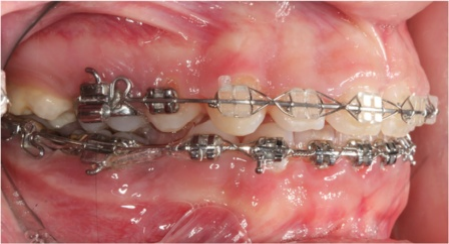
Orthodontic space closure using a retraction elastic chain to close the space between the permanent first molar and the first premolar; a stop was added to the wire to maximize anchorage

Ankylosis of the mesial root of the primary mandibular right second molar was diagnosed after 10 weeks through clinical and radiographic examination, and the splint was removed (Fig. 4). Next, a band was placed on the permanent mandibular right first molar and brackets were bonded on the primary second molar and the mandibular first premolar. A closing T-loop 0.017″ × 0.025″ TMA sectional wire was inserted between the permanent and primary molar, without any prior levelling, to move the permanent first molar mesially (Fig. 5). The T-loop was activated 9 times. At 6 months, clinical and radiographic examination revealed almost total replacement resorption of the root of the primary tooth and complete closure of the space (Fig. 6). Consequently, the bisected primary tooth was extracted and full orthodontic treatment followed. Remaining space closure was performed, during the next year, using elastic chain on a 0.017″ × 0.025″ SS wire with tip-back (Fig. 7) and support from class II elastics.

**Fig. 8:**
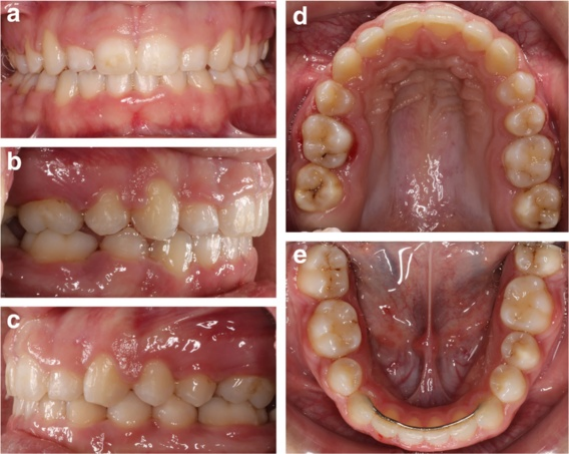
Clinical examination after the completion of orthodontic treatment showing the 1st permanent molar in the position of the 2nd premolar **a** frontal view, **b** right lateral view, **c** left lateral view, **d** maxillary occlusal view, and **e** mandibular occlusal view

**Fig. 9:**
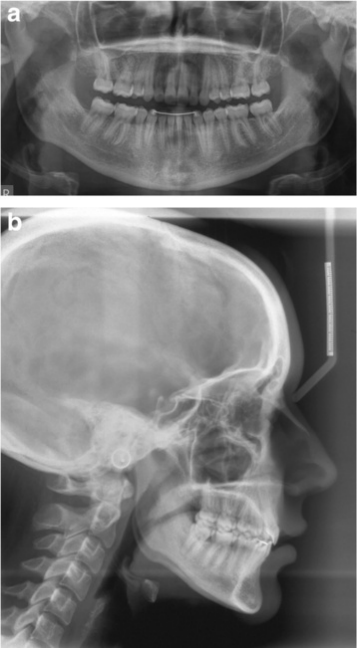
**a** Panoramic radiograph at the end of orthodontic treatment showing the 1st permanent molar in the position of the 2nd premolar. **b** Final cephalometric radiograph

Final records show class I canine and molar relationship on both sides; full space closure of the agenesis sites, confirmed with the use of dental floss; and correction of overbite and overjet; however, a slight midline deviation remained (Figs. 8 and 9a). Cephalometric analysis showed that the mandibular incisors did not experience lingual movement as a result of molar protraction (Fig. 9b, Table 1).

### Discussion

Induced ankylosed primary teeth can be used in place of temporary anchorage devices to maximize anchorage during orthodontic treatment. The technique used in the present case was based on the treatment protocol for avulsed teeth exceeding 60 min extraoral dry time, as suggested by the International Association of Dental Traumatology [11]. However, the tooth was not placed in 2 % sodium fluoride for 20 min since the goal of this step is to postpone osseous replacement of the root [10], which was not a desired outcome in this case. Also, a rigid splint was used to further assure tooth ankylosis [12]. Previous studies that have reported induced ankylosis for orthodontic protraction have used similar techniques [7–9].

Several treatment alternatives have been proposed for the management of congenital missing premolars. In cases of deep bite, spacing, and class I occlusion, the primary tooth can be kept in place until it exfoliates [2, 13]. In cases of congenitally missing premolars, the primary predecessors usually exfoliate when the patient is an adult [13, 14] and an implant can be placed, as the alveolar bone has usually been preserved [2, 15]. However, it is difficult to restore the implant since the mesiodistal dimension of the primary tooth is greater than the missing premolar and additional orthodontic treatment may be required [2, 14, 16].

An option to avoid future restorative compromise, especially when orthodontic treatment is inevitably required, is to modify the primary molar [2] by trimming it mesially and distally and restoring it as a premolar [2]. Orthodontic treatment follows and, when the primary molar exfoliates, it is replaced by an implant [2].

When the primary molar has extensive caries, restorations, or resorption, extraction is an option [1, 14]. The space can then be preserved with a space maintainer or an implant placed if the patient is an adult [2, 3, 15]. In all implant cases mentioned above, a fixed prosthesis is also an alternative [3]. It is crucial to note that when space is maintained for a long time, alveolar bone can become atrophic and implant placement is challenging [2, 4]. Finally, autotrasplantation of premolars or molars may also be an alternative [17].

In cases of crowding, class II malocclusion, severely damaged primary molars, or when the patient rejects the option of a prosthesis, space closure is the treatment of choice [1, 2]. When the patient is under the age of 8 years, spontaneous space closure can be achieved [18, 19]. The key point is to detect the premolar agenesis prior to root completion of the first permanent molar [18, 19]. However, even then, mild tipping of adjacent teeth can be observed [18, 19].

An alternative to space closure is the use of conventional orthodontics. In our case, this was the choice of treatment for the space closure in the maxilla, where cortical alveolar bone is less compact and thus orthodontic bodily movement is easier [20]. However, in the mandible, due to the bone’s compact architecture, bodily movement is compromised leading to loss of anchorage and undesired tooth movement [3, 5, 20,21]. Lingual functional appliances [22], intermaxillary elastics [4, 16], and mandibular protraction appliances [21] have been proposed for space closure in the mandible to avoid molar tipping and anchorage loss.

Another option is controlled slicing and hemisectomy [14, 23]. Controlled slicing starts with progressive trimming of the distal surface of the primary molar which leads to hemisectomy of the tooth [14]. The hemisectomy technique is similar to the one used for induced ankylosis; however, the primary molar is not ankylosed and cannot be used as an immovable abutment. Hemisectomy diminishes the risk of bone atrophy in the extraction site and of extreme mesial inclination of the first permanent molar [14, 23]. However, undesired movement of the anterior teeth cannot be totally prevented [23].

Recently, orthodontic mini-implants have been used successfully for space closure in mandibular molar protraction [2, 3, 6, 24]. However, they have the risk of soft tissue inflammation, damage of anatomical structures during implant placement, lack of stability, and implant fracture [25–27]. On the contrary, an induced ankylosed tooth has maximum biocompatibility leading to low inflammation risks when appropriate oral hygiene is performed. Furthermore, there is no risk to damage anatomical structures during replantation since the tooth is put in the existing socket. Finally, the root of the ankylosed tooth at the time of final extraction has almost totally been replaced by bone diminishing the risk of fracture.

The proposed technique is conservative and has advantages since (a) half of the tooth is retained, thereby retaining the alveolar process during the first phase of protraction, and (b) even if ankylosis fails to develop, other options are still open, because the tooth can be extracted and treatment can continue using other methods (e.g., mini-implant). However, this technique is indicated only for cooperative patients since procedures are time-consuming and treatment requires several visits. In addition, treatment cost can be high compared to mini-implants. Also, the ankylosed abutment cannot be placed wherever is required but where the primary tooth is located and is only partially exploited since the ankylosed root has to be extracted after 4–5 mm of protraction.

## Abbreviations

TMA: Titanium molybdenum alloy
SS: Stainless steel
